# Childhood Personality Assessment Q-Sort (CPAP-Q): A Clinically and Empirically Procedure for Assessing Traits and Emerging Patterns of Personality in Childhood

**DOI:** 10.3390/ijerph18126288

**Published:** 2021-06-10

**Authors:** Alexandro Fortunato, Annalisa Tanzilli, Vittorio Lingiardi, Anna Maria Speranza

**Affiliations:** Department of Dynamic and Clinical Psychology, and Health Studies, “Sapienza”, University of Rome, Via Degli Apuli, 1, 00185 Rome, Italy; annalisa.tanzilli@uniroma1.it (A.T.); vittorio.lingiardi@uniroma1.it (V.L.); annamaria.speranza@uniroma1.it (A.M.S.)

**Keywords:** child personality, diagnosis, emerging personality patterns, CPAP-Q

## Abstract

Background: Despite increasing research confirming the existence of childhood personalities, which are recognizable from a developmental perspective, controversies over the possibility to assess personality in childhood have continued. The purpose of this study was to provide initial data on the validation of the Childhood Personality Assessment Q-Sort (CPAP-Q), a clinician report instrument that can be employed to evaluate children’s personalities and address the gap in the field of emerging personality in children classification. Method: A sample of 135 clinicians completed the CPAP-Q to assess the personality features of 135 children (ages 4–11) who had been in their care between two and 12 months. The clinicians completed a clinical questionnaire to collect information on them, the children, and their families, as well as the Child Behavior Checklist (CBCL), to evaluate the children’s behavioral problems and social competencies. Results: Q-factor analysis identified seven specific emerging personality patterns: psychological health, borderline/impulsive, borderline/dysregulated, schizoid, inhibited/self-critical, obsessive, and dysphoric/dependent. These patterns revealed good levels of validity and reliability. Conclusions: These findings are preliminary, but seem to support the possibility of evaluating emerging personality patterns in childhood and their developmental pathways that may lead to personality disorders in adolescence and adulthood. The CPAP-Q promises to significantly contribute to less explored research areas and encourage systematic studies of children assessment, promoting best practices for individualized diagnoses.

## 1. Introduction

International scientific debate on personality pathologies in childhood, as well as the appropriate clinical and diagnostic system for their classification, is still characterized by several controversies [1,2,3]. Concerns, calls for caution, and more or less temperate admonitions of the Diagnostic and Statistical Manual of Mental Disorders (most recently, DSM-IV-TR and DSM-5; [4,5]) against personality diagnosis during childhood development are linked to three specific issues. First, personality related to age-specific changes in children’s mental functioning, including ways of organizing subjective experience, relating to others, facing difficulties, and adapting to external environment, lacks stability. Second, it is difficult to distinguish the typical characteristics of children’s normal psychological development from signs and symptoms of several psychopathological syndromes that are optimal for adults, but not necessarily for children. Finally, the risk of stigma associated with personality diagnoses is meaningful, and brings up ethical considerations [6,7,8,9].

Presently, an increasing body of cross-sectional and longitudinal empirical research, based on extended theoretical literature and the extensive clinical work of authors such as P. Kernberg et al. [10], Bleiberg [11], and Greenspan et al. [12], has revealed the existence of personality patterns that are recognizable in childhood [1,13,14,15,16,17,18,19,20,21].

Although childhood is a fluid, dynamic, and changeable developmental period, research has shown that specific traits and patterns of perception, relationship, and thought that have emerged from the interaction of genetic and psychosocial factors tend to be relatively stable. These personality patterns organize and structure the quality of children’s internal experience, as well as their defensive and coping strategies, relatedness, cognitive processes, and adaptation [22,23,24,25,26,27,28,29,30,31]. If personality traits and configurations become rigid and persistent, it makes sense to assume the presence of an emerging personality pattern that requires clinical attention and early intervention planning in order to prevent negative mental health outcomes.

Research during the last 20 years has revealed that enduring and maladaptive personality traits may be assessed in childhood, are not reducible to other clinical conditions, such as conduct problems, attention deficit hyperactivity disorder, oppositional defiant disorder, and depressive and anxiety disorders, and may predict several personality diagnoses and psychopathological syndromes in adolescence and adulthood [32,33,34,35]. For example, various studies have found that impulsiveness in preschool children and behavioral problems in school-aged children were strongly related to antisocial behaviors in subsequent development periods [36,37,38,39], or could be predictive of borderline personality disorder [40,41]. Similarly, withdrawal, inhibition, and shyness in early childhood may be significant precursors to avoidant personality traits in adolescence [35].

In line with the *Developmental Psychopathology* approach [42,43] and especially with the perspective of homotypic and heterotypic psychopathological continuity (i.e., the fact that one disorder predicts the same or another disorder at a later point [44,45]), many authors recognize that, despite differences in directly observable features, signs, and symptoms, the maturational and psychopathological processes underpinning some disorders are similar. Identifying developmental trajectories of psychopathological configurations by considering the child’s personality and its core developmental dynamics enables more specific diagnostic predictions and promotes more timely interventions [46]. Overall, the need to put a greater attention on detection and early identification of personality characteristics and patterns in childhood seems to have become urgent. The lack of accurate and developmentally oriented diagnoses may expose children to several dangers and striking long-term consequences, with an increased risk of developing severe clinical conditions in adolescence or adulthood [47]. The importance of adapting the diagnostic process to specific life stages and assuming a developmental perspective has been emphasized in the new edition of the Psychodynamic Diagnostic Manual (PDM-2 [46,48,49]). It is noteworthy that the PMD-2 is strongly grounded in the wide corpus of clinical knowledge and the results of empirical investigations in the field of diagnosis and assessment. Furthermore, the manual proposes the assessment of personality in childhood, taking into account the fluidity typical of development processes and experiential changes during this period in life [50].

However, the PDM-2 does not classify personality patterns of children, but is limited to describing their personality organizations (healthy, neurotic, borderline, and psychotic). This gap is partially due to the lack of systematic and extensive research on personality patterns in childhood. It is important to note that how to classify emerging personality patterns in children and whether adult personality disorders may be employed in developmental stages are debatable. Moreover, the inadequate number of children’s personality assessment instruments represents another relevant problem. Although clinical and diagnostic interviews could be useful techniques, children may not be able to understand all of the questions. Even though projective tests can also be employed to assess the presence and severity of features of personality pathology in children, information and evaluations of different constructs, such as reality testing, disturbance of thought, cognitive functioning, affects, and representations of self and others are imperative. Other tools, such as cognitive and behavioral scales, comprise many items that correspond to personality features and traits that can be applied to the diagnosis. While some of these items reflect particular personality disorders, others reveal personality characteristics in general. However, these tools are problematic in that one cannot make a diagnosis with them, and they focus exclusively on symptomatology [21,48].

Among the possible informants, the clinician may be considered to be one of the most trustworthy. Accordingly, the Q-Sort method is used mainly to investigate adolescence and adulthood personality. The Shedler–Westen Assessment Procedure-200, which was developed by Westen and Shedler [51,52,53,54,55], is the most popular Q-Sort method to assess personality. Notably, the SWAP-200-A [56,57,58,59], the adolescent version, may be utilized to verify that personality disorders in adolescence are present and to identify a specific taxonomy of personality configurations in this developmental stage that are empirically derived.

### 1.1. Development and Features of the Childhood Personality Assessment Procedure Q-Sort (CPAP-Q)

A literature review [7] demonstrated the lack of systematic research on childhood personality and a paucity of tools that may be employed to indicate the developmental paths of personality. It is crucial that an instrument for evaluating childhood personality should not only understand personality pathology, which may be rarer during development, but also possible maladaptive trajectories.

Accordingly, a number of years ago, we decided to develop a tool inspired by the SWAP-200, which would consider the validation studies for adults and adolescents [51,52,57,59]. It must be specified that the ultimate goal of this Q-Sort was not only to identify childhood personality disorders where present, but also emerging patterns. Our aim was to create an instrument that could be employed to identify key aspects that would be useful for a complete evaluation by focusing on childhood personality as a construct that includes all aspects of children’s functioning.

Four personality organizations and eight emerging personality patterns were derived from a theoretical and clinical review. Although a brief description follows, refer to Fortunato and Speranza [7] for a complete description of the patterns. While the first personality organization comprises the healthy personality, the second consists of the neurotic personality organization, and includes inhibited/withdrawn, pathological obsessiveness, and dysphoric emerging personality patterns. The third, the borderline personality organization, includes dysregulated and pathological narcissism emerging personality patterns, and the fourth, the psychotic personality organization, comprises suspicious and schizoid emerging personality patterns.

Through the four domains of the mental functions defined by the PDM-2, namely cognitive and affective processes, identity and relationships, defense and coping, and self-awareness and self-direction [48], we derived the characteristics of each pattern and developed the 200 items of the CPAP-Q that describe specific configurations of affect, cognition, motivation, and behavior in childhood. Beyond the notion of whether it is better not to label, avoiding stigmatization, and allowing development to follow its own course, or rather be at risk for traits that exist that lead to a structured pathology without intervention, we are of the view that emerging personality patterns in childhood as developmental trajectories that can be examined should be considered.

We are of the opinion that an examination of the precursors and pathways of personality disorders during childhood, integrating developmental issues, biological vulnerabilities, and problematic environments, has the potential to define a longitudinal developmental approach to personality development and psychopathology. Our approach is an endeavor to address this challenge by conjugating both top-down (theoretical) and bottom-up (research) perspectives, and is based on research and clinical evidence [7].

### 1.2. Aims

The purpose of this study was to address the gap in the research on children’s personality assessments and provide a new assessment procedure for classifying emerging personality patterns in childhood. This research focused on two primary goals. The first goal was to employ the CPAP-Q to develop an empirically grounded, psychometrically robust, and clinically useful system for diagnosing emerging personality pathology in children. The second goal was to furnish preliminary data on the CPAP-Q’s validity and reliability by assessing the associations between empirically derived personality patterns and various variables of the adaptive functioning and constellation of symptoms in a clinical population of children. In accordance with clinical and empirical literature in the field, the following two hypotheses were tested.

**Hypothesis** **1.** *In order to identify naturally occurring grouping empirically among child patients on the basis of their personality configurations and in accordance with previous studies on adolescent populations [57,59], we hypothesized that specific groups of patients have personality features similar within their own grouping and distinct from those in other patient groupings.*

**Hypothesis** **2.** *In order to verify the reliability and validity of this empirically derived classification to evaluate emerging personality pathologies in children, we hypothesized that this classification is grounded in specific diagnostic categories and criteria that assess children’s personalities in developmentally sound and clinically sophisticated ways. In line with the clinical and research literature in the field, we hypothesized that there are significant differences in all of the empirically derived personality dimensions between children in relation to various demographic and developmental variables, such as gender and early traumatic experiences, as well as distinct DSM-5 [5] psychiatric diagnoses. Moreover, these personality categories are significantly associated with children’s specific social and behavioral problems, as well as internalizing and externalizing symptomatology in a well-differentiated and clinically coherent manner.*

## 2. Materials and Methods

### 2.1. Participant Sampling

A practice network approach was employed to recruit an Italian sample of experienced clinicians and collect data of children in their care. The membership rosters of the Italian associations of developmental psychotherapy and centers that specialize in the treatment of children were utilized to contact clinicians via e-mail. The clinicians’ group was selected from among mental health professionals who reported treating children and had at least three years of post-psychotherapy licensure experience. They agreed to participate in a research project on psychological assessment in childhood. All of the clinicians provided informed written consent to participate in the research project.

### 2.2. Procedure

To obtain a broad range of personality patterns, the clinicians were asked to describe a child patient they were treating or evaluating who displayed enduring and maladaptive patterns of thought, feeling, motivation, or behavior (i.e., personality problems) that caused distress or dysfunction. They were specifically directed to select a child randomly in accordance with the following inclusion/exclusion criteria: (a) between four and 11 years of age; (b) no psychotic psychiatric disorder based on the DSM-5 [5] classification system and not treated with drug therapy for psychotic symptoms; (c) no traumatic brain injury, neurological disorder, and/or clinically significant cognitive impairment; (d) no autistic spectrum disorders; and (e) in treatment between two and 12 months. To minimize the bias associated with patient selection, the clinicians were asked to consult their calendars and provide data on the last patient they had seen the previous week who met the study criteria. The patients were not involved in any way in the research, and the clinicians received no remuneration. The overall response rate was approximately 15%.

### 2.3. Measures

The battery of instruments employed in this research project on children’s personalities included several questionnaires and assessment procedures. The most relevant measures are subsequently described.

Clinical Questionnaire: We constructed a questionnaire for clinicians to acquire general information about themselves, their patients, and therapies. The clinicians provided their basic demographic data, including their age, gender, race, and profession, namely psychiatrist or psychologist, years of experience, and theoretical orientation. They also provided information on their patients’ demographic, diagnostic, developmental, and family history. Specifically, they reported information about the children’s traumatic experiences or events, including neglect and mistreatments, parental abandonment or early separations, and therapies, such as length of treatments.

Child Behavior Checklist–Clinician Version (CBCL, 4–18; [60]): The CBCL is a questionnaire designed to assess children’s behavioral and emotional difficulties and social competencies in order to investigate a broad spectrum of children and adolescent developmental characteristics. The CBCL evaluates the behavior through two scales of internalizing and externalizing symptomatology. It comprises 128 items, which are grouped in 11 problem scales and four competence scales. The clinicians completed a clinician report version of the parent form of this tool. High levels of validity and reliability that are similar to those of the parent and teacher report versions have been found for this version [60,61].

Childhood Personality Assessment Procedure-Q sort (CPAP-Q; [62]): The CPAP-Q is a clinician report procedure that is employed to assess emerging personality patterns in childhood. It is based on a Q-Sort method that is utilized in the context of personality pathology and psychological functioning of children. The CPAP-Q comprises 200 statements or items, which the clinician or rater is required to sort into eight categories. These categories range from those that are irrelevant or not descriptive of the patient (assigned a value of 0) to those that are highly descriptive (assigned a value of 7). Intermediate categories include statements that may apply in varying degrees. The 200 items are formulated by using simple, atheoretical, and non-jargon language, and describe all eight prototypes of emerging personality patterns and other clinical conditions, including sleep, feeding, and neurodevelopment.

After the CPAP-Q was constructed, the instrument was subjected to a preliminary validation through a theoretical and statistical consensus to test the items and patterns. The theoretical consensus was obtained by employing the services of 30 specialist clinicians that evaluated each pattern and item on a five-point Likert scale. While all patterns were rated 4 or 5, most of the items were rated similarly, and only a few were rated 3. The statistical consensus involved 42 clinicians who assessed 42 children (M = 7.92; 64% male). We evaluated the mean score and standard deviation (SD) for each item. Only 16 of the 200 items had an SD lower than 1.50 and a small mean score. Thereafter, Cronbach’s alpha was performed to evaluate each pattern’s internal coherence. The results revealed that good or excellent levels for each emerging pattern were obtained (healthy personality α = 0.88, inhibited/withdrawn α = 0.91, pathological obsessiveness α = 0.92, dysphoric α = 0.90, dysregulated α = 0.94, pathological narcissism α = 0.94, suspicious α = 0.84, schizoid α = 0.92).

Prototypes have had an excellent outcome for both theoretical and statistical consensus, as well as that of the items. In line with the consensus results, we modified the problematic items to obtain the final version of the instrument. However, the prototypes are not used in the same manner as the personality disorder (PD) scales of the SWAP [51,52,53,57,59,63], but only as basic components of the tool, because there is no unanimous agreement on the possibility of diagnosing personality disorders during development, and thus, the prototypes remain a theoretical proposal [7].

The statements of the CPAP-Q are expressed in a simple manner, which resembles clinical experience, so that the instrument can be used comparably by therapists of any orientation. Moreover, standard vocabulary with which clinicians may express their clinical judgments and observations is employed, which allows clinicians to provide the specific personality configurations of patients that can be quantified and compared with psychological descriptions furnished by other mental health professionals, as well as analyzed statistically.

The Q-Sort normally requires clinicians or raters to assign a certain number of items to each category in accordance with the fixed distribution of the Q-Sort method [64]. In line with the Shedler–Westen Assessment Procedure for adults and adolescents [51,52,56,59], the clinicians were asked to use a semi-constrained procedure, which required a fixed distribution of the most descriptive categories of the CPAP-Q, including categories 5, 6, and 7 [57]. The rationale of this choice was to verify whether the rigid constraints of a fixed distribution developed for adults and adolescents would be appropriate for child populations. Moreover, this approach maximized the participants’ rate of response given that the Q-Sort’s standard sorting procedure is more time-consuming and articulated.

### 2.4. Statistical Analyses

To identify naturally occurring diagnostic groupings empirically, a particular form of factor analysis, the Q-factor analysis or Q-analysis, was performed on the CPAP-Q’s data provided by clinicians. Whereas conventional factor analyses serve to group specific sets of variables of a common underlying trait or dimension, the Q-analysis is a statistical technique that identifies groups of similar people who are characterized by a common syndrome or configuration of symptoms that distinguishes them from other groups. In the present study, all 200 items in the CPAP-Q were utilized in the Q-analysis to determine similarities in a child clinical population, and thus develop configurations of personalities that consider broadband psychological domains, such as affective regulation, defense mechanisms, interpersonal functioning, cognitive capacities, identity aspects, and resources and strengths.

To obtain an empirically derived classification of personality patterns in childhood, Q-analysis on the CPAP-Q’s data, as well as a principal component analysis (PCA; cfr., [51,52,57]) with promax rotation because of our expectation of correlated Q-factors, were performed. To select the number of Q-factors to be extracted and rotated, Kaiser’s criteria eigenvalues > 1, the scree plot, replicability across other estimation procedures and rotations (e.g., unweighted least squares with promax rotation; see, for example, [59]), and percentage of variance accounted for by the factor solution and its interpretability were considered.

After identifying diagnostic groupings (Q-factors), we created dimensional scores for each patient (Q-scores), which reflect the degree of fit between the child’s 200-item personality configuration and each Q-factor or empirically derived prototype. Subsequently, to assess the initial validity of the diagnostic prototypes, the Mann–Whitney U test was performed to verify differences in all of the empirically derived personality dimensions between the children in relation to gender, early traumatic experiences, such as presence/absence of grief, separation, abandonment, and neglect, and specific DSM-5 psychiatric diagnoses, including presence/absence of anxiety disorders, depressive disorders, disruptive, impulse control, and conduct disorders. Furthermore, bivariate correlations (Pearson’s r, two-tailed) were performed to examine the associations among the Q-scores of the CPAP-Q’s personality dimensions and the child’s age, behavioral and interpersonal problems, and internalizing and externalizing symptomatology, which were assessed with the CBCL–Clinician Version. Finally, we constructed paragraph length diagnostic prototypes of child personality pattern scales that could be used in everyday practice.

## 3. Results

### 3.1. Clinicians’ Characteristics

The sample comprised 135 clinicians, specifically 85 (63%) women and 50 (37%) men; there were 21 (15%) clinical psychologists, 94 (70%) psychotherapists, and 20 (15%) neuropsychiatrists. The principal theoretical and clinical approaches represented included psychodynamic/psychoanalytic (N = 78), cognitive/behavioral (N = 28), systemic/relational (N = 5), integrated (N = 17), and other (N = 7). The psychotherapists’ average clinical experience was 11.25 years (SD = 8.3; range = 3–35). The average length of treatments was 6.73 months (SD = 3.54; range = 2–12). The results further revealed that 70% and 30% of the patients described were from private settings and public mental health services in the national territory, respectively.

### 3.2. Patients’ Characteristics

The clinical sample included 135 patients, namely 43 (31.9%) females and 92 males (68.1%). The children’s average age was 8.7 years (SD = 1.66; range 4–11). Of the 135 patients, 98 (73%) had specific DSM-5 [5] psychiatric diagnoses, including disruptive, impulse control, and conduct disorders (DICD; 9.2%), anxiety disorders (20.4%), specific learning disorders (SLD; 9.2%), depressive disorders (9.2%), attention deficit hyperactivity disorders (ADHD; 16.3%), obsessive-compulsive disorder (DOC; 6.1%), and suspected autism spectrum disorder (ASD; 7.1%). The diagnoses of the other 22.5% included sleep–wake disorders, motor disorders, communication disorders, and evacuation disorders.

While the mothers’ average age was 41.27 years (SD = 5.23; range 27–58), that of the fathers was 43.41 years (SD = 6.17; range 30–70). Furthermore, approximately 73% were married or cohabiting and 27% were separated or divorced. Moreover, 64% of the children had siblings.

### 3.3. Deriving an Empirically Based Classification of Child Emerging Personality Patterns: Q-Factors

To develop an empirically based taxonomy of children’s emerging personality patterns, we conducted a Q-analysis of the CPAP-Q data provided by 135 clinicians about their patients. Considering the Kaiser–Meyer–Olkin score of 0.74 and Bartlett’s test of sphericity, χ^2^ (9045) 0 21342.59, *p* < 0.001, we performed a PCA. This procedure accounted for approximately 49% of the variance, and resulted in seven Q-factors, which ranged from 16.70% to 2.50%. Although they were subjected to promax rotation, the same dimensions that had emerged using other extraction procedures followed by different rotations emerged. This solution was the most clinically coherent, because all seven Q-factors were readily interpretable and psychometrically robust, including those of patients with factor loadings of 0.50 or higher.

The CPAP-Q items that described all of the patients included in each empirically derived diagnostic prototype are presented in Table 1, Table 2, Table 3, Table 4, Table 5, Table 6 and Table 7. The seven Q-factors were labeled psychological health index, borderline/impulsive, borderline/dysregulated, schizoid, inhibited/self-critical, obsessive, and dysphoric/dependent. It is noteworthy that the Q-analysis was conducted on a clinical population of children who were identified theoretically and clinically with consistent diagnostic categories. While some of the Q-factors (e.g., schizoid and obsessive) resembled current personality pathologies that have emerged in adolescent and adult samples [52,57,59,63], others represented important constellations of personality traits and features that occur naturally in child patients.

Overall, the seven configurations of personality took into account a broad range of psychological dimensions, including affect and impulse regulation, interpersonal functioning, cognitive capacities, representations of self and others, and resources. The first prototype describes child patients with clinically relevant strengths, and reflects a useful index of global personality functioning in childhood.

The two borderline prototypes represent two configurations that share the difficulty of controlling impulses and acting in emotionally distressing situations. While the borderline/impulsive prototype describes children characterized by several psychopathic traits, such as hostility, lack of remorse, incapacity for intimate relationships, and deceitfulness, the borderline/dysregulated prototype describes children with strong and poorly modulated emotions, fear of abandonment, outbursts of anger, and unstable relationships that are characterized by abrupt fluctuations between over-involvement and withdrawal.

The schizoid prototype is characterized by restricted affectivity, social detachment and emotional aloofness, lack of empathy, eccentricity, and bizarre behaviors. On the contrary, the inhibited/self-critical prototype describes shy and reserved children who avoid social situations, are inhibited, passive, and unassertive, feel inadequate or inferior, and do not enjoy close friendships.

The obsessive prototype comprises personality traits of rigid perfectionism, need for control, fear of criticism, constricted experiences, expression of strong emotion, and intimacy avoidance. Finally, the dysphoric/dependent prototype describes children that are anxious, overly needy, dependent, display depressivity and unhappiness, and experience separation insecurity, especially when their feelings about intimate involvement with their parents are centered around a strong fear of rejection and abandonment.

To verify the internal consistencies of all CPAP-Q’s factors, we calculated Cronbach’s alpha and found that all were above 0.80: psychological health, α = 0.93; borderline/impulsive, α = 0.95; borderline/dysregulated, α = 0.90; schizoid, α = 0.92; inhibited/self-critical, α = 0.88; obsessive, α = 0.88; and dysphoric/dependent, α = 0.87.

The comparison between the emerging personality patterns that were identified theoretically from the literature review [7] and the Q-factors that were derived empirically are displayed in Table 8. While similarities between some personality patterns and specific Q-factors were found in some cases, there were significant differences in others. Where there was a correspondence (psychological health, obsessive, and schizoid), the differences were found in the hierarchy of the items. In those where there were some differences, new traits were added (inhibited/self-critical and dysphoric/dependent). The two borderline factors, which were theoretically similar in the patterns, but substantially different in the items, were distinguished by the characteristics of dysregulation and impulsivity, thus providing important diagnostic information to distinguish not only the types of childhood personalities, but also different developmental patterns.

### 3.4. Validity of the Empirically Derived Taxonomy

To perform the initial test of validity of the diagnostic system of Q-factors from the CPAP-Q, differences in distinct patients’ personality dimensions in relation to several demographic variables and diagnostic categories were tested by employing the Mann–Whitney U test.

No significant gender differences were found in the levels of all personality dimensions. In relation to all of the other variables, the results depicted in Table 9 revealed significant differences in the borderline/impulsive personality dimension between patients with and without ADHD. Specifically, the scores of borderline/impulsive personality were greater for ADHD patients than for those without this diagnosis. Significant differences were found in the borderline/dysregulated personality dimension between patients with and without early traumatic experiences. The scores of borderline/dysregulated personality were higher for patients who had suffered traumatic events in early childhood than for those who had not encountered such.

Furthermore, the findings revealed differences in the schizoid personality dimension between patients with and without suspected ASD. It is noteworthy that the scores of schizoid personality were significantly greater for patients who presented with suspected ASD than for all of the other patients. Moreover, children with and without suspected ASD differed significantly in the psychological health personality dimension. In fact, patients with suspected ASD scored lower in this dimension than other ones.

There were also significant differences in the inhibited/self-critical personality dimension between the patients with and without depressive disorders. Children with depressive disorders scored higher for this dimension than those without these clinical conditions. Significant differences were also found in the dysphoric/dependent personality dimension between patients with and without anxiety disorders. The patients with anxiety disorders scored higher on this dimension than those without these diagnoses.

Finally, significant differences were found in the obsessive personality dimension between patients with and without DOC. In particular, the patients with DOC scored higher than those without this diagnosis.

To provide a further test of validity, we evaluated the associations between the children’s personality profiles (Q-scores), age, and various scales of the CBCL. These latter criterion variables are particularly significant, given that they currently allow for the assessment of maladaptive patterns of thoughts, emotions, motivations, and behaviors in child clinical samples.

First, the children’s age was significantly related to the psychological health dimension. The older children tended to display greater psychological strength and inner resources that afford a more enhanced adaptation to their environmental context. In Table 10, the strong convergence and divergence is presented. As expected, while the borderline/impulsive dimension correlated significantly with CBCL delinquent and aggressive behaviors, the schizoid dimension correlated with withdrawn and social problems. Psychological health correlated negatively with total CBCL problems. In general, it is noteworthy that, while the borderline/impulsive and borderline/dysregulated dimensions were significantly related to CBCL externalizing, the other emerging personality patterns were related to CBCL internalizing.

## 4. Discussion

The purpose of the present study was to provide a new measure to assess children’s personalities from a clinician’s perspective. Consistent with our first hypothesis, the results seem to support CPAP-Q as a useful instrument to evaluate emerging personality patterns in childhood. Seven groups of child patients were identified on the basis of a broad spectrum of cognitive, affective, and behavioral dimensions. This new taxonomy includes seven personality categories and diagnostic criteria, and offers an approach to diagnose children’s emerging personalities in an empirically grounded, psychometrically robust, and clinically meaningful way.

The seven personality prototypes that correspond to the conceptually coherent and clinically sensitive personality dimensions are psychological health, borderline/impulsive, borderline/dysregulated, schizoid, inhibited/self-critical, obsessive, and dysphoric/dependent. The preliminary data on the reliability support that each prototype has an excellent internal consistency. Various considerations on childhood personality and its continuity through these prototypes can be made. It is noteworthy that, in accordance with the literature, some personalities show homotypic continuity over time from childhood to adolescence and adulthood. These include obsessive [65,66,67], schizoid [68,69,70,71], and those characterized by psychological health [52,57,59].

The inhibited/self-critical and the dysphoric/dependent prototypes concur with similar personality dimensions identified in clinical adolescent and adult populations that have employed the SWAP-200 and SWAP-200-A [57,59]. Notably, the depressive personality, which is still absent in official nosography, exists and tends to be persistent and on a continuum in various stages of development [72].

With regard to the borderline/dysregulated and borderline/impulsive prototypes, both belong to the borderline area. However, if children are characterized more by their dysregulation, they are likely to be more predisposed to borderline and histrionic personality disorders in adulthood [73,74,75,76,77,78]. On the contrary, those known for their impulsivity tend to suffer narcissistic and antisocial disorders [10,36,79,80,81,82,83,84]. It is likely that the different trajectories will emerge in adolescence when they encounter a pubescent body and their peer group has an influence on them. This is a very clinically useful difference that allows us to grasp the nuances of personality in childhood, which is not as evident as those in adults.

A factor related to suspicion and diffidence is unknown, possibly because of the rarity of this condition, particularly in childhood [48]. This did not emerge in the small sample of this study, but may do so in a larger sample.

With regard to the second goal of the study, we found that the CPAP-Q had good validity and reliability by assessing the associations between prototypes and some variables and diagnoses. The link between the borderline/impulsive prototype and ADHD concurs with the literature [41,76,83], as well as the relationship between the borderline/dysregulated prototype and early traumatic experiences [84,85,86,87,88].

It is noteworthy how children with suspected ASD fall largely within the schizoid personality. These data could lead the way to future developments and reflections on how the diagnosis of autism is often used as a container for many different situations, and may not necessarily be related to the autism spectrum [89]. It is possible that investigating children’s personality may help clinicians to clarify this diagnosis by eliminating all of the complex and difficult situations from investigations on the field of autism, which, due to a lack of personality diagnosis, are included in the only diagnostic category that can currently contain them.

Consistent with our expectations, depressive symptoms, DOC, and anxiety problems are associated with the inhibited/self-critical, obsessive, and dysphoric/dependent personalities, respectively.

The correlations between the children’s personality profiles (Q-scores) and some scales of the CBCL (Table 10) revealed strong convergence and divergence validity. The associations between the prototypes and syndromic scales concurs with the clinical literature. It is notable that psychological health was negatively correlated with almost all of the syndromic scales, with the exception of the anxious/depressed scale. A possible explanation for this exception is that all of the children in the sample, even those with a highly functioning personality, were clinical patients in treatment for some form of developmental maladjustment. The high negative correlation between this factor and total problems is particularly meaningful. The inhibited/self-critical and dysphoric/dependent prototypes have shown strong correlations with anxious/depressed, withdrawn, somatic complaints, as well as social problems, which concurs with the literature [35,90,91,92,93,94,95]. As expected, they were also related to internalizing problems and thought and attention problems, which is consistent with neurodevelopment difficulties.

The obsessive prototype revealed significant correlations with anxiety, depression, withdrawal, and internalizing problems. This concurs with the literature that has noted how these children experience daily activities and relationships, and are dominated by these emotions [64,65,66].

The borderline/dysregulated and borderline/impulsive prototypes revealed a high correlation with social and attention problems, aggressive and delinquent behavior, and externalizing problems. This concurs with the literature [11,37,41,79,83], as well as the description of the prototypes.

The schizoid prototype was highly correlated with withdrawal, social, thought, and attention problems. This is consistent with the description of the factor and with research that has highlighted the level of impairment of this type of personality [70].

## 5. Conclusions

The data presented are preliminary, but promising. A much larger sample is necessary to confirm the taxonomy of emerging personality patterns from the CPAP-Q. However, initial data on psychometric properties of this instrument and its dimensions have shown good criterion validity and high levels of reliability. Thus, one may deduce that the CPAP-Q is useful to study childhood personality.

A first classification of personality, even if only verified at the end of this study, contributes to the debate on whether one can evaluate personality and its disorders in childhood. One may conclude that one can evaluate personality and its disorders in childhood, particularly continuity and risk factors. The prototypes allow us to trace evolutionary, non-deterministic trajectories on which it is possible to intervene in advance. An understanding of this aspect may be deepened through longitudinal studies.

Therefore, it appears that, even in children, the Q-Sort procedure is the most suitable for evaluating personality, because it addresses clinicians and affords them the opportunity to think. A tool such as the CPAP-Q obliges those who employ it to reflect on all of the possible features of patients’ functioning, to reflect on what has not yet been observed, providing opportunities to think and offering an entire vision of children. One of our primary goals was to establish the clinical benefits of the instrument. One may deduce that the tool could be employed to evaluate the effectiveness of therapeutic interventions and changes that occur.

Clinicians’ assessment of personality is the actual strength of this procedure, even if, methodologically, it does not allow one to have informants, which would be desirable. Accordingly, the procedure does not interfere with the treatment or in the relationship with patients and their parents. This allows one to access a great deal more data while, at the same time, preserving the therapeutic relationship.

## 6. Limitations

The two main limitations of this study were the small sample and the use of a single informant. In relation to the former, it is important to highlight that these data are preliminary, and, therefore, it is recommended that larger samples should be used in further studies. Furthermore, future research should examine children’s personalities by employing other measurement methods and perspectives, such as an independent observer. However, previous research has suggested that clinicians tend to make very reliable and valid judgments if their observations and inferences are quantified by employing psychometrically sophisticated instruments, such as those used in this study [51,52,96,97].

## Figures and Tables

**Table 1:** Best CPAP-Q ^a^ items of the psychological health prototype.

Items ^b^	Factor Score
Tends to use age-appropriate language	6.20
Tends to finish what she/he has begun	5.87
Tends to appreciate and respond to humor	5.80
Tends to use talents, skills, and energy in an effective and productive way	5.53
Tends to be conscientious and responsible	5.53
Tends to be creative	5.20
Tends to be liked by others	5.20
Tends to have relationships based on intimacy and closeness; has best friends	4.87
Tends to be empathetic, sensitive, and responsive to the needs and feelings of others	4.87
Tends to feel at ease in social situations	4.80
Tends to feel satisfied and happy about what she/he is doing and experience a sense of validity	4.80
Tends to let others console her/him when faced with negative situations	4.73
Tends to be happy on her/his own	4.73
Tends to express affects that are appropriate to the situation in quality and intensity	4.73
Tends to have high moral and ethical standards	4.73
Tends to be dynamic or expansive	4.40
Tends to be able to cope with stress or stressful situations and conflicts with appropriate feelings (in relation to the nature and intensity of the situation)	4.33
Tends to be very polite, respecting social norms and conventions (excessively so)	4.33
Tends to be psychologically intuitive; is able to understand herself/himself and others	4.20
Tends to enjoy challenges; enjoys achieving goals	4.07

^a^ Childhood Personality Assessment Procedure-Q sort [62]. ^b^ Items presented in descending order of importance.

**Table 2:** Best CPAP-Q ^a^ items of the borderline/impulsive prototype.

Items ^b^	Factor Score
Tends to be conflictual, difficult, or ready to disagree	6.70
Tends not to consider the consequences of her/his behavior and actions	6.60
Tends to get into power struggles with adults	6.50
Tends to be impulsive or to act without thinking	6.50
Tends to be inflexible, stubborn, sulky, or irritable	6.00
Tends to be disobedient at home and at school	6.00
Tends to feel angry and conflictual (both overtly and covertly)	6.00
Tends to resort to violence or intimidation to control someone deemed important (e.g., siblings, parents)	5.90
Tends to get easily frustrated (e.g., gives up easily)	5.70
Tends to shout a lot	5.70
Tends to have difficulty maintaining friendships	5.70
Tends to blame others for her/his own failings or flaws; tends to believe her/his problems are caused by external factors	5.70
Tends to lose interest or be easily distracted; has problems with concentration	5.60
Behavior at school is erratic, unpredictable, or extremely inappropriate (e.g., is lazy or extremely destructive in class)	5.50
Tends to destroy her/his own or other people’s things and be aggressive	5.50
Tends to lie or cheat	5.50
Tends to be unreliable and irresponsible (e.g., does not do homework)	5.50
Tends not to abide by rules laid down by others	5.50
Tends to perceive criticism even when there is none and is always ready to react	5.50
Tends not to get along well with peers	5.50

^a^ Childhood Personality Assessment Procedure-Q sort [62]. ^b^ Items presented in descending order of importance.

**Table 3:** Best CPAP-Q ^a^ items of the borderline/dysregulated prototype.

Items ^b^	Factor Score
Tends to spiral out of control, leading to extreme anxiety, unhappiness, anger, excitement, etc.	5.67
Tends to establish various forms of reciprocal control relationships with adults (e.g., victim–aggressor, victim–rescuer, or some other caricature-like role)	5.67
Tends to be subject to sudden mood swings or emotional shifts	5.44
Tends to express emotions in an extravagant and dramatic way	5.33
Tends to be afraid of being rejected or abandoned by people who are emotionally important to her/him	5.22
Tends to express anger in a way that is disproportionate to the situation	5.11
Tends to feel angry and conflictual (both overtly and covertly)	5.11
Tends to get easily frustrated (e.g., gives up easily)	4.78
Tends to be impulsive or act without thinking	4.78
Tends to provoke intrusive and controlling or hostile and detached parenting behavior	4.56
Tends to be very attentive to how adults react; is sensitive to other people’s moods	4.44
Tends to create processes of role reversal with a parent in response to the parent’s inability to take care of her/him	4.44
Tends to manipulate other people’s feelings in order to obtain what she/he wants	4.44
Tends not to view adults as being protective	4.44
She/he has past experiences of trauma, neglect, various kinds of abuse, or major stress	4.44
Tends to provoke feelings in others that are similar to those that she/he is feeling (e.g., when angry, she/he acts in a way that arouses anger in others; when anxious, she/he acts in a way that makes others anxious; when feeling helpless, she/he makes others feel helpless)	4.44
Her/his family history is marked by severe disorders	4.33
Tends to arouse extreme reactions and strong feelings in others	4.33
Tends to develop somatic symptoms in response to stress or conflict (e.g., headache, backache, stomach-ache, asthma, enuresis, encopresis, etc.)	4.22
Tends to need others, but at the same time reject them (e.g., continually demands attention, but then tends to reject it)	4.22

^a^ Childhood Personality Assessment Procedure-Q sort [62]. ^b^ Items presented in descending order of importance.

**Table 4:** Best CPAP-Q ^a^ items of the schizoid prototype.

Items ^b^	Factor Score
Tends to have a very limited range of affects	6.80
Tends to have neither close relationships nor friends	6.50
Tends to resort to magical thinking, fantasy, and strange ideas (inappropriate for her/his age)	6.17
Tends to behave in strange ways	6.17
Tends to be shy, timid, and withdrawn, especially in social situations	6.17
Tends to have poor social skills; in social situations, tends to behave awkwardly and inappropriately	5.83
Tends to display a worsening of her/his usual functioning, to the extent that previously acquired skills are lost	5.67
Speech tends to be tortuous, vague, disconnected, full of digressions, etc.	5.67
Tends to think in concrete terms and interpret things in an excessively literal way; is not very skilled at appreciating metaphors, analogies, or shades of meaning (for her/his age)	5.67
Tends to lose interest or be easily distracted. Has problems with concentration	5.50
Tends to play in a way that is not age appropriate (e.g., games are compulsive, inflexible, not enjoyable, non-elaborate, frequently interrupted, empty and dull)	5.50
Tends to have set rituals linked to urinating or defecating, going to bed, or eating	5.33
Tends to get easily frustrated (e.g., gives up easily)	5.33
Learned to walk and/or talk later than other children	5.33
Tends to lack empathy (has difficulty understanding and appreciating other people’s ideas, feelings, or behavior)	5.33
Tends to be fairly uncoordinated, clumsy, and awkward	5.17
Tends to be ignored, neglected, or avoided by her/his peers	5.00
Tends to have difficulty maintaining friendships	5.00
Tends to describe experiences in general terms; does not want or is not able to provide details (or does so in a way that is age inappropriate)	5.00
Tends to be fairly inarticulate; is not able to express herself/himself well with words	5.00

^a^ Childhood Personality Assessment Procedure-Q sort [62]. ^b^ Items presented in descending order of importance.

**Table 5:** Best CPAP-Q ^a^ items of the inhibited/self-critical prototype.

Items ^b^	Factor Score
Tends to feel ashamed or embarrassed	6.33
Tends to be passive and not very assertive	5.67
Tends to be anxious	5.50
Tends to be inhibited or subject to coercion; has trouble recognizing or expressing her/his own desires and impulses	5.50
Tends to think of herself/himself as having little value	5.50
Tends to worry about being criticized, disapproved of, or rejected in social situations	5.50
Tends to be afraid of being rejected or abandoned by people who are emotionally important to her/him	5.50
Tends to be shy, timid, and withdrawn, especially in social situations	5.50
Tends to be indecisive or very uncertain when faced with choices	5.33
Tends to feel bored, unhappy, depressed, and dejected	5.33
Tends to be self-critical; sets unrealistically high standards for her/himself, and cannot tolerate her/his own personal flaws	5.17
Tends to find little or no pleasure, satisfaction, or enjoyment in everyday activities	5.00
Tends to have difficulty feeling strong pleasurable emotions (e.g., excitement, joy, pride)	5.00
Tends to be inhibited by the prospect of achieving goals or by success in general (e.g., is afraid to take center stage or put ideas forward at school)	5.00
Tends to feel inadequate, inferior, or incompetent (both overtly and covertly)	5.00
Tends to have poor social skills; in social situations, tends to behave awkwardly and inappropriately	5.00
Tends to be very sensitive to criticism at school	4.83
Her/his family history is marked by severe disorders	4.83
Tends to be reticent; keeps things to herself/himself	4.83
Tends to feel impotent and at the mercy of others (e.g., parents)	4.67

^a^ Childhood Personality Assessment Procedure-Q sort [62]. ^b^ Items presented in descending order of importance.

**Table 6:** Best CPAP-Q ^a^ items of the obsessive prototype.

Items ^b^	Factor Score
Tends to be anxious	6.25
Tends to be very polite, respecting social norms and conventions (excessively so)	6.25
Tends to be worried, have false expectations, or be dissatisfied with school results	6.25
Tends to be hypervigilant and controlling	6.25
Tends to worry about being criticized, disapproved of, or rejected in social situations	6.25
Tends to waste a lot of time doing things the way she/he thinks they should be done; a perfectionist at the expense of flexibility, open-mindedness, and efficiency	6.25
Tends to be competitive (both overtly and covertly)	6.25
Tends to be very sensitive to criticism at school	6.00
Tends to be afraid of getting angry; does not want to appear aggressive (e.g., freezes at moments of intense emotion)	6.00
Tends to worry too much about tidiness and cleanliness (e.g., after a fall, goes to wash her/himself straightaway)	6.00
Tends to express aggression in passive and indirect ways (e.g., sulking)	5.75
Tends to be inhibited or subject to coercion; has trouble recognizing or expressing her/his own desires and impulses	5.75
Tends to be self-critical; sets her/himself unrealistically high standards and cannot tolerate her/his own personal flaws	5.75
Tends to be afraid of being rejected or abandoned by people who are emotionally important to her/him	5.75
Tends to be conscientious and responsible	5.75
Tends to swing from being very dependent and needy to being very independent and avoidant	5.50
Tends to stick rigidly to daily routine and gets anxious or feels uncomfortable if it changes	5.50
Tends to be embarrassed by displays of affection. Does not like effusive behavior, closeness, and intimacy (e.g., hugs and kisses)	5.25
Tends to feel ashamed or embarrassed	5.25
Tends to be excessively worried about rules, practices, order, organization, and scheduling; dislikes losing control of her/his environment and affects	5.25

^a^ Childhood Personality Assessment Procedure-Q sort [62]. ^b^ Items presented in descending order of importance.

**Table 7:** Best CPAP-Q ^a^ items of the dysphoric/dependent prototype.

Items ^b^	Factor Score
Tends to be anxious	6.50
Tends to be very sensitive to criticism at school	6.25
Tends to be needy or over-dependent (e.g., demanding excessive reassurance or approval, being over-attached to friends and parents)	6.00
Tends to be afraid of everything, consistent with the anxieties and fears of her/his parents	6.00
Tends to be excessively anxious socially, and this does not decrease with familiarity	6.00
Tends to have difficulties with reading, writing, and arithmetic or generally with learning anything new	5.75
Tends to have problems with food (e.g., too little or too much, selective eating, etc.)	5.75
Tends to display a worsening of her/his usual functioning, to the extent that previously acquired skills are lost	5.75
Tends to have panic attacks lasting from a few minutes to several hours, accompanied by intense physical reactions (e.g., elevated heart rate, shortness of breath, a choking sensation, nausea, and dizziness)	5.50
Tends to use her/his own medical or psychological issues as an excuse not to attend school or face up to responsibilities (both overtly and covertly)	5.50
Tends to do anything, even offering to perform unpleasant tasks, in order to obtain protection and support from others	5.25
Tends to feel ashamed or embarrassed	5.00
Tends to develop somatic symptoms in response to stress or conflict (e.g., headache, backache, stomach-ache, asthma, enuresis, encopresis, etc.)	5.00
Tends to get easily frustrated (e.g., gives up easily)	5.00
Tends to be fairly uncoordinated, clumsy, and awkward	5.00
Tends to feel uncomfortable at school, in social situations, or anywhere outside the home	5.00
Tends to feel inadequate, inferior, or incompetent (both overtly and covertly)	4.75
Tends to feel that parents only bestow care when she/he is ill	4.75
Tends not to start or carry out tasks alone for fear of being laughed at or mocked	4.75
Tends to feel powerless, weak, or at the mercy of forces beyond her/his control	4.75

^a^ Childhood Personality Assessment Procedure-Q sort [62]. ^b^ Items presented in descending order of importance.

**Table 8:** Comparison between emerging personality patterns and Q-factors.

Emerging Personality Patterns ^a^	Q-Factors ^b^
Healthy Personality	Psychological Health
Inhibited/Withdrawn	Inhibited/Self-critical
Pathological Obsessiveness	Obsessive
Dysphoric	Dysphoric/Dependent
Dysregulated	Borderline/Dysregulated
Pathological Narcissism	Borderline/Impulsive
Suspicious	
Schizoid	Schizoid

^a^ Emerging personality patterns: derived from literature review [7]. ^b^ Q-factors: diagnostic prototypes empirically derived.

**Table 9:** Differences of CPAP-Q ^a^ personality prototypes in relation to specific demographic and diagnostic variables.

	Groups	N	*Mdn*	Mann–Whitney *U* Test	*Z*	*p* (Two-Tailed)
Psychological Health	Suspected ASD	7	1.48	127.50	−3.18	≤0.001
	No Suspected ASD	128	3.05			
Borderline/Impulsive	ADHD	16	4.00	533.50	−2.85	0.004
	No ADHD	119	2.14			
Borderline/Dysregulated	Early Traumatic Experiences	57	3.84	1527.50	−3.10	0.002
	No Early Traumatic Experiences	78	3.00			
Schizoid	Suspected ASD	7	5.57	37	−4.08	≤0.001
	No Suspected ASD	128	2.43			
Inhibited/Self-Critical	Depressive Disorders	9	5.00	272	−2.60	0.009
	No Depressive Disorders	126	3.00			
Obsessive	DOC	6	4.50	189	−2.18	0.029
	No DOC	129	3.00			
Dysphoric/Dependent	Anxiety Disorders	20	3.83	780	−2.29	0.022
	No Anxiety Disorders	115	3.10			

^a^ Childhood Personality Assessment Procedure-Q sort [62].

**Table 10:** Correlations between CPAP-Q ^a^ personality prototypes and CBCL ^b^ scales.

	Withdrawn	Somatic Complaints	Anxious/Depression	Social Problems	Thought Problems	Attention Problems	Delinquent Behavior	Aggressive Behavior	Total Problems	Internalizing	Externalizing
Psychological Health	−0.26 **	−0.07	0.06	−0.44 ***	−0.19 *	−0.57 ***	−0.38 ***	−0.43 ***	−0.49 ***	−0.11	−0.38 ***
Borderline/Impulsive	0.15	0.05	0.07	0.60 ***	0.27 **	0.64 ***	0.76 ***	0.81 ***	0.75 ***	0.01	0.79 ***
Borderline/Dysregulated	0.11	0.17 *	0.32 ***	0.47 ***	0.25 **	0.35 ***	0.56 ***	0.69 ***	0.64 ***	0.10	0.67 ***
Schizoid	0.51***	0.12	0.08	0.73 ***	0.49 ***	0.71 ***	0.42 ***	0.16	0.61 ***	0.19 *	0.12
Inhibited/Self-Critical	0.60 ***	0.25 **	0.67 ***	0.39 ***	0.24 **	0.12	−0.02	−0.05	0.36 ***	0.66 ***	−0.04
Obsessive	0.37 ***	0.18 *	0.55 ***	0.16	0.13	−0.17 *	−0.12	−0.15	0.12	0.43 ***	−0.16
Dysphoric/Dependent	0.34 ***	0.37 ***	0.61***	0.47 ***	0.20 *	0.19 *	0.16	0.07	0.42 ***	0.47 ***	0.08

^a^ Childhood Personality Assessment Procedure-Q sort [62]. ^b^ Child Behavior Checklist—Clinician Version. * *p* ≤ 0.05. ** *p* ≤ 0.01. *** *p* ≤ 0.001.

## Data Availability

Not Applicable.

## References

[1] Caspi A., Roberts F., Shiner R. (2005). Personality development: Stability and change. Annu. Rev. Psychol..

[2] McAdams D.P., Olson B.D. (2010). Personality Development: Continuity and change over the life course. Annu. Rev. Psychol..

[3] Widiger T.A., De Clercq B., De Fruyt F. (2009). Childhood antecedents of personality disorder: An alternative perspective. Dev. Psychopathol..

[4] American Psychiatric Association (2000). Diagnostic and Statistical Manual of Mental Disorders, DSM IV-R..

[5] American Psychiatric Association (2013). Diagnostic and Statistical Manual of Mental Disorders.

[6] Cicchetti D., Crick N.R. (2009). Precursors and diverse pathways to personality disorder in children and adolescents. Dev. Psychopathol..

[7] Fortunato A., Speranza A.M. (2018). Personality traits and disorders in childhood: Clinical evaluation and diagnosis. Clin. Neuropsychiatry J. Treat. Eval..

[8] Shapiro T. (1990). Debate forum-Resolved: Borderline personality disorder exists in children under twelve. J. Am. Acad. Child. Adolesc. Psychiatr..

[9] Magnavita J.J., Levy K.N., Critchfield K.L., Lebow J.L. (2010). Ethical considerations in treatment of personality dysfunction: Using evidence, principles, and clinical judgment. Prof. Psychol. Res. Pract..

[10] Kernberg P., Weiner A., Bardenstein K. (2000). Personality Disorders in Children and Adolescents.

[11] Bleiberg E. (2001). Treating Personality Disorders in Children and Adolescents. A Relational Approach.

[12] Greenspan S.I., Hatleberg J.L., Cullander C.C. (1976). A systematic metapsychological assessment of the personality in childhood. J. Am. Psychoanal. Assoc..

[13] Bernstein D., Cohen P., Velez N., Schwab-stone M., Siever L., Shinsato L. (1993). Prevalence and stability of the DSM-III personality disorder in a community-based survey of adolescents. Am. J. Psychiatry.

[14] De Clercq B., DeFruyt F. (2012). A five factor model framework for understanding childhood personality disorder antecedents. J. Personal..

[15] De Clercq B., Van Leeuwen K., Van Den Noortgate W., De Bolle M., DeFruyt F. (2009). Childhood personality pathology: Dimensional stability and change. Dev. Psychopathol..

[16] Edmonds G.W., Goldberg L.R., Hampson S.E., Barckley M. (2013). Personality stability from childhood to midlife: Relating teachers’ assessments in elementary school to observer and self-relating 40 years later. J. Res. Personal..

[17] Golombeck H., Marton P., Stein B., Korenblum M. (1986). Personality dysfunction and behavioral disturbance in early adolescence. J. Am. Acad. Child. Psychiatr..

[18] Shiner R.L. (2009). The development of personality disorders: Perspectives from normal personality development in childhood and adolescence. Dev. Psychopathol..

[19] Shiner R.L., Caspi A. (2003). Personality di¡erences in childhood and adolescence: Measurement, development, and consequences. J. Child. Psychol. Psychiatry.

[20] Roberts B.W., Del Vecchio W.F. (2000). The rank-order consistency of personality traits from childhood to old age: A quantitative review of longitudinal studies. Psychol. Bull..

[21] Tackett J.L. (2010). Measurement and assessment of child and adolescent personality pathology: Introduction to the special issue. J. Psychopathol. Behav. Assess..

[22] Beeghly M., Cicchetti D. (1994). Child maltreatment, attachment, and the self system: Emergence of an internal state lexicon in toddlers at high social risk. Dev. Psychopathol..

[23] Cicchetti D., Rogosch F.A. (1996). Equifinality and multifinality in developmental psychopathology. Dev. Psychopathol..

[24] Cicchetti D., Toth S. (1995). A developmental psychopatology perspective on child abuse and neglect. J. Am. Acad. Child. Adolesc. Psychiatry.

[25] Fonagy P. (2000). Attachment and borderline personality disorder. J. Am. Psychoanal. Assoc..

[26] Fonagy P., Target M. (1997). Attachment and reflective function: Their role in self-organization. Dev. Psychopathol..

[27] Perry B.D., Pollard R. (1998). Homeostasis, stress, trauma, and adaptation: A neurodevelopmental view of childhood trauma. Child. Adolesc. Psychiatr. Clin. N. Am..

[28] Rutter M. (1987). Temperament, personality, and personality disorder. Br. J. Psychiatry.

[29] Rutter M. (1999). Resilience concepts and findings: Implications for family therapy. J. Fam. Ther..

[30] Sroufe L.A. (1997). Emotional Development: The Organization of Emotional Life in the Early Years.

[31] Wyman P.A., Cowen E.L., Work W.C., Hoyt-Meyers L., Magnus K.B., Fagen D.B. (1999). Caregiving and developmental factors differentiating young at-risk urban children showing resilient versus stress-affected outcomes: A replication and extension. Child Dev..

[32] Kagan J., Zentener M. (1996). Early childhood predictors of adult psychopathology. Harv. Rev. Psychiatry.

[33] Caspi A., Harrington H., Milne B., Amell J.W., Theodore R.F., Moffitt T.E. (2003). Children’s behavioral styles at age 3 are linked to their adult personality traits at age 26. J. Personal..

[34] De Clercq B., Verbeke L., De Caluwé E., Vercruyss E.T., Hofmans J. (2017). Understanding adolescent personality pathology from growth trajectories of childhood oddity. Dev. Psychopathol..

[35] Eggum N.D., Eisenberg N., Spinrad T.L., Valiente C., Edwards A., Kupfer A.S., Reiser M. (2009). Predictors of withdrawal: Possible precursors of avoidant personality disorder. Dev. Psychopathol..

[36] Bergman L.R., Andershed H., Andershed A.K. (2009). Types and continua in developmental psychopathology: Problem behaviors in school and their relationship to later antisocial behavior. Dev. Psychopathol..

[37] Calkins S.D., Keane S.P. (2009). Developmental origins of early antisocial behavior. Dev. Psychopathol..

[38] Lahey B.B., Moffitt T.E., Caspi A. (2003). Causes of Conduct Disorder and Juvenile Delinquency.

[39] Moffitt T.E., Lahey B.B., Moffitt T.E., Caspi A. (2003). Life-course-persistent and adolescence-limited antisocial behavior: A 10-year research review and a research agenda. Causes of Conduct Disorder and Juvenile Delinquency.

[40] Caspi A. (2000). The child is father of the man: Personality continuities from childhood to adulthood. J. Personal. Soc. Psychol..

[41] Stepp S.D., Burke J.D., Hipwell A.E., Loeber R. (2012). Trajectories of attention deficit hyperactivity disorder and oppositional defiant disorder symptoms as precursors of borderline personality disorder symptoms in adolescent girls. J. Abnorm. Psychol..

[42] Cicchetti D., Cohen D.J. (2006). Developmental Psychopathology: Theory and Method.

[43] Rutter M., Sroufe L.A. (2000). Developmental psychopathology: Concepts and challenges. Dev. Psychopathol..

[44] Costello E.J., Angold A., Chicchetti D., Choen D.J. (1995). Developmental epidemiology. Developmental Psychopathology: Volume 1. Theory and Methods.

[45] Costello E.J., Mustillo S., Erkanli A., Keeler G., Angold A. (2003). Prevalence and development of psychiatric disorders in childhood and adolescence. Arch. Gen. Psychiatry.

[46] Speranza A.M., Malberg N., Steele M. (2018). Mental health and developmental disorders in infancy and early childhood: The PDM-2. Psychoanal. Psychol..

[47] Greenspan S.I., Wieder S. (2006). Infant & Early Childhood Mental Health: A Comprehensive Developmental Approach to Assessment and Intervention.

[48] Lingiardi V., McWilliams N. (2017). Psychoanalytic Diagnostic Manual. Second Edition (PDM-2).

[49] Malone J.C., Piacentini E., Speranza M. (2018). Reclaiming the developmental lens for adolescent formulation and diagnosis: Application of the PDM-2 to Clinical Cases. Psychoanal. Psychol..

[50] Lingiardi V., McWilliams N. (2015). The Psychodynamic Diagnostic Manual–2nd ed. (PDM-2). World Psychiatry.

[51] Westen D., Shedler J. (1999). Revising and assessing Axis II, Part I: Developing a clinically and empirically valid assessment method. Am. J. Psychiatry.

[52] Westen D., Shedler J. (1999). Revising and assessing Axis II, Part II: Toward an empirically based and clinically useful classification of personality disorders. Am. J. Psychiatry.

[53] Shedler J., Westen D., Lingiardi V. (2014). La Valutazione Della Personalità con la SWAP-200.

[54] Shedler J., Westen D. (2004). Dimensions of personality pathology: An alternative to the five-factor model. Am. J. Psychiatry.

[55] Tanzilli A., Lingiardi V., Hilsenroth M. (2018). Patient SWAP-200 personality dimensions and FFM traits: Do they predict therapist responses?. Personal. Disord. Theory Res. Treat..

[56] Westen D., Chang C., Esman A.H., Flaherty L.T., Horowitz H.A. (2000). Personality pathology in adolescence: A review. The Annals of the American Society for Adolescent Psychiatry. Adolescent psychiatry: Developmental and Clinical Studies.

[57] Westen D., Shedler J., Durrett C., Glass S., Martens A. (2003). Personality diagnoses in adolescence: DSM-IV axis II diagnoses and an empirically derived alternative. Am. J. Psychiatry.

[58] Tanzilli A., Gualco I. (2020). Clinician emotional responses and therapeutic alliance when treating adolescent patients with narcissistic personality disorder subtypes: A clinically meaningful empirical investigation. J. Personal. Disord..

[59] Westen D., DeFife J.A., Malone J.C., DiLallo J. (2014). An empirically derived classification of adolescent personality disorders. J. Am. Acad. Child. Adolesc. Psychiatry.

[60] Achenbach T. (1991). Manual for the Child Behavior Checklist/4-18 and 1991 Profile.

[61] Achenbach T.M., Rescorla L.A. (2000). Manual for ASEBA Preschool Forms and Profiles.

[62] Fortunato A., Speranza A.M., Tanzilli A., Lingiardi V. (2018). CPAP-Q: Childhood Personality Assessment Q-Sort.

[63] Westen D., Shedler J., Bradley B., DeFife J.A. (2012). An empirically derived taxonomy for personality diagnosis: Bridging science and practice in conceptualizing personality. Am. J. Psychiatry.

[64] Block J. (1978). The Q-Sort Method in Personality Assessment and Psychiatric Research.

[65] Andrews G., Stewart G., Morris-Yates A., Holt P., Henderson S. (1990). Evidence for a general neurotic syndrome. Br. J. Psychiatry.

[66] Stone M.H. (1993). Long-term outcome in personality disorders. Br. J. Psychiatry.

[67] Parker Z., Stewart E. (1994). School consultation and the management of obsessive-compulsive personality in the classroom. Adolescence.

[68] Olin S.C.S., Raine A., Cannon T.D., Parnas J., Schulsinger F., Mednick S.A. (1997). Childhood behavior precursors of schizotypal personality disorder. Schizophr. Bull..

[69] Seivewright H., Tyrer P., Johnson T. (2002). Change in personality status in neurotic disorders. Lancet.

[70] Lenzenweger M.F., Willett J.B. (2009). Does change in temperament predict change in schizoid personality disorder? A methodological framework and illustration from the Longitudinal Study of Personality Disorders. Dev. Psychopathol..

[71] Wolff S., Townshend R., McGuire R.J., Weeks D.J. (1991). ‘Schizoid’ personality in childhood and adult life. II: Adult adjustment and the continuity with schizotypal personality disorder. Br. J. Psychiatry.

[72] Rudolph K.D., Klein D.N. (2009). Exploring depressive personality traits in youth: Origins, correlates, and developmental consequences. Dev. Psychopathol..

[73] Berenbaum H., Raghavan C., Le H.N., Vernon L.L., Gomez J.J. (2003). A taxonomy of emotional disturbances. Clin. Psychol. Sci. Pract..

[74] Cicchetti D., Ackerman B.P., Izard C.E. (1995). Emotions and emotion regulation in developmental psychopathology. Dev. Psychopathol..

[75] Cole P.M., Llera S.J., Pemberton C.K. (2009). Emotional instability, poor emotional awareness, and the development of borderline personality. Dev. Psychopathol..

[76] Cole P.M., Michel M.K., Teti L.O.D. (1994). The development of emotion regulation and dysregulation: A clinical perspetive. Monogr. Soc. Res. Child Dev..

[77] Gross J.J., Muñoz R.F. (1995). Emotion regulation and mental health. Clin. Psychol. Sci. Pract..

[78] Kring A.M., Werner K.H., Philippot P., Feldman R.S. (2004). Emotion regulation and psychopathology. The Regulation of Emotion.

[79] Campbell S.B. (1995). Behavior problems in preschool children: A review of recent research. J. Child Psychol. Psychiatry.

[80] Pettit G.S., Bates J.E., Dodge K.A. (1993). Family interaction patterns and children’s conduct problems at home and school: A longitudinal perspective. Sch. Psychol. Rev..

[81] Caye A., Swanson J., Thapar A., Sibley M., Arseneault L., Hechtman L., Arnold L.E., Niclasen J., Moffitt T.E., Rohde L.A. (2016). Life Span Studies of ADHD—Conceptual Challenges and Predictors of Persistence and Outcome. Curr. Psychiatry Rep..

[82] Moffitt T.E. (2018). Male antisocial behaviour in adolescence and beyond. Nat. Hum. Behav..

[83] Wertz J., Agnew-Blais J., Caspi A., Danese A., Fisher H.L., Goldman-Mellor S., Moffitt T.E., Arseneault L. (2018). From childhood conduct problems to poor functioning at age 18 years: Examining explanations in a longitudinal cohort study. J. Am. Acad. Child. Adolesc. Psychiatry.

[84] Sharp C., Tackett J.L. (2014). Handbook of Borderline Personality Disorder in Children and Adolescents.

[85] Agrawal H.R., Gunderson J., Holmes B.M., Lyons-Ruth K. (2004). Attachment studies with borderline patients: A review. Harv. Rev. Psychiatry.

[86] Herman J., Russell D., Trocki K. (1986). Long-term effects of incestuous abuse in childhood. Am. J. Psychiatry.

[87] Carlson E.A., Egeland B., Sroufe L.A. (2009). A prospective investigation of the development of borderline personality symptoms. Dev. Psychopathol..

[88] Crawford T.N., Cohen P.R., Chen H., Anglin D.M., Ehrensaft M. (2009). Early maternal separation and the trajectory of borderline personality disorder symptoms. Dev. Psychopathol..

[89] Kulage K.M., Smaldone A.M., Cohn E.G. (2014). How Will DSM-5 Affect Autism Diagnosis? A Systematic Literature Review and Meta-analysis. J. Autism Dev. Disord..

[90] Boone M.L., McNeil D.W., Masia C.L., Turk C.L., Carter L.E., Ries B.J., Lewin M.R. (1999). Multimodal comparisons of social phobia subtypes and avoidant personality disorder. J. Anxiety Disord..

[91] Marteinsdottir I., Tillfors M., Furmark T., Anderberg U.M., Ekselius L. (2003). Personality dimensions measured by the Temperament and Character Inventory (TCI) in subjects with social phobia. Nordic J. Psychiatry.

[92] Rettew D.C. (2000). Avoidant personality disorder, generalized social phobia, and shyness: Putting the personality back into personality disorders. Harv. Rev. Psychiatry.

[93] LaFreniere P. (2009). A functionalist perspective on social anxiety and avoidant personality disorder. Dev. Psychopathol..

[94] Meyer B. (2002). Personality and mood correlates of avoidant personality disorder. J. Personal. Disord..

[95] Warner M.B., Morey L.C., Finch J.F., Gunderson J.G., Skodol A.E., Sanislow C.A., Shea M.T., McGlashan T.H., Grilo C.M. (2004). The longitudinal relationship of personality traits and disorders. J. Abnorm. Psychol..

[96] Blagov P.S., Bi W., Shedler J., Westen D. (2012). The Shedler-Westen Assessment Procedure (SWAP): Evaluating psychometric questions about its reliability, validity, and impact of its fixed score distribution. Assessment.

[97] Westen D., Weinberger J. (2004). When clinical description becomes statistical prediction. Am. Psychol..

